# Diagnostic Criteria of Postoperative Cognitive Dysfunction: A Focused Systematic Review

**DOI:** 10.1155/2020/7384394

**Published:** 2020-11-16

**Authors:** Kim van Sinderen, Lothar A. Schwarte, Patrick Schober

**Affiliations:** Department of Anesthesiology, Amsterdam University Medical Centers, Vrije Universiteit Amsterdam, Amsterdam, Netherlands

## Abstract

Postoperative Cognitive Dysfunction (POCD) is characterized by a deterioration in cognitive performance after surgery and is increasingly addressed in research studies. However, a uniform definition of POCD seems to be lacking, which is a major threat to clinical research in this area. We performed a focused systematic review to determine the current degree of heterogeneity in how POCD is defined across studies and to identify those diagnostic criteria that are used most commonly. The search identified 173 records, of which 30 were included. Neurocognitive testing was most commonly performed shortly before surgery and at 7 days postoperatively. A variety of neurocognitive tests were used to test a range of cognitive domains, including complex attention, language, executive functioning, perceptual-motor function, and learning and memory. The tests that were used most commonly were the Mini-Mental State Examination, the digit span test, the trail making test part A, and the digit symbol substitution test, but consensus on which test result would be considered “positive” for POCD was sparse. The results of this systematic review suggest the lack of a consistent approach towards defining POCD. However, commonalities were identified which may serve as a common denominator for deriving consensus-based diagnostic guidelines for POCD.

## 1. Introduction

Postoperative Cognitive Dysfunction (POCD) is characterized by a deterioration in cognitive performance after surgery and is particularly prevalent in elderly patients [1]. The cognitive decline is usually transient, but can be a substantial threat to the quality of life as it occasionally persists for months to years after surgery. The pathophysiology and risk factors are incompletely understood, and effective strategies for prevention and treatment yet need to be established. Thus, POCD is the target of an increasing amount of research studies and reviews published in leading anesthesia journals [2–4].

Despite the clinical relevance and the need for standardized research, a uniform definition of POCD seems to be lacking. A systematic review in 2010 revealed a major inconsistency in diagnostic criteria, neurocognitive tests, and timing of the assessment of POCD across studies in cardiac surgery [5]. The need for uniform terminology and diagnostic criteria in POCD research has in the meantime been widely recognized, and recommendations on uniform nomenclature for perioperative neurocognitive disorders have been published [6]. Yet, it is unclear whether POCD research has recently adopted a more uniform approach to defining POCD and which criteria and tests are currently most commonly used to define POCD.

We thus performed a focused systematic review, limited to literature published in the last two years, to determine the current degree of heterogeneity in how POCD is defined and diagnosed across research studies. The results of this review may identify those tests and criteria that are currently used most often and can serve as a common denominator to derive a broader consensus on which tests and criteria should actually be used to diagnose POCD.

## 2. Methods

PubMed was searched for original research studies on POCD in adult patients published between January 1, 2018 and January 14, 2020, using the following search strategy: (“Postoperative Cognitive Complications” [Mesh] OR “Postoperative Period” [Mesh] OR Postoperative [tiab] OR Post-operative [tiab]) AND (“Cognitive Dysfunction” [Mesh] OR (cognitive dysfunction [tiab] OR cognitive dysfunctioning [tiab] OR cognitive dysfunctions [tiab]) OR cognitive decline [tiab]) AND ((“2018/01/01” [PDAT]: “2020/01/14” [PDAT]) AND “adult” [MeSH Terms]). Titles and abstracts were screened by 2 authors, and the full text of potentially relevant articles was retrieved. Original research studies on human subjects, published in English language, that explicitly reported which diagnostic criteria had been used to define POCD, were eligible for inclusion. Data on study and patient characteristics, POCD criteria, and tests used to assess POCD, as well as the time points at which the tests were performed, were extracted using a standardized data extraction form.

Because the research question does not lend itself to quantitative pooling of data across studies, and due to substantial heterogeneity across studies, a meta-analysis of the collected data was not performed, and all results are presented descriptively.

## 3. Results

**Figure 1:**
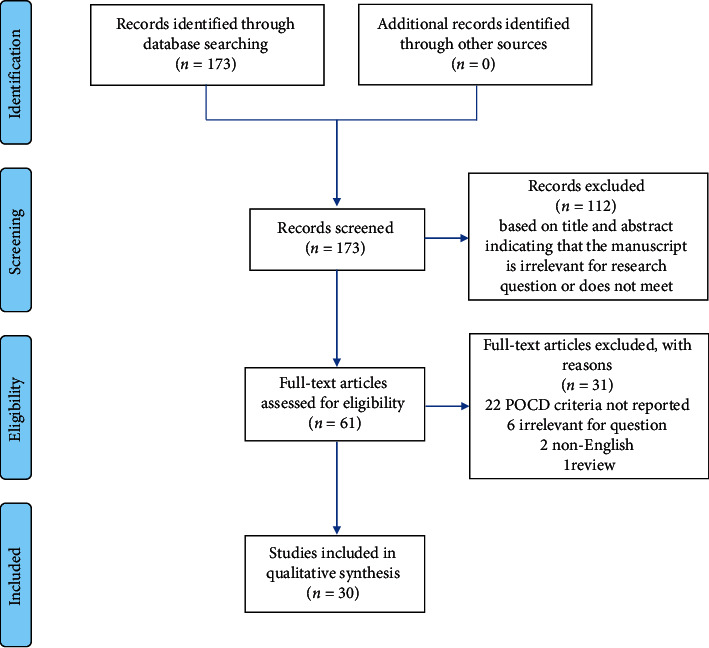
Flow diagram of studies included in the systematic review.

The PubMed search identified 173 records, and 61 full-text articles were assessed for eligibility (Figure 1). Of these, 30 studies reporting POCD criteria were included in this systematic review [7–36]. Seven studies report data of a randomized controlled trial, while the rest were observational study designs (cohort, case-control, and longitudinal studies). The study characteristics, POCD criteria, and POCD assessment tests and time points are summarized in Table 1.

In the majority of studies (*n* = 25), baseline neurocognitive performance was measured either on the day of surgery or on the day before surgery, while the other studies used varying preoperative time intervals. The most frequently used follow-up time was 7 days after surgery (*n* = 23). Ten out of the 30 studies followed patients up over a longer period of time, up to one year after surgery.

A variety of different neurocognitive tests (Table 2) were used to test a range of cognitive domains, including complex attention, language, executive functioning, perceptual-motor function, and learning and memory. We refer to Sachdev et al. [37] for a detailed description of the different cognitive domains and to Evered and Silbert [3] for an overview over which domains are being tested by which neurocognitive test. Nine studies included in our systematic review relied on only one test, either the Mini-Mental State Examination (MMSE, *n* = 5) or the Montreal Cognitive Assessment (MoCA, *n* = 4), both of which actually test several neurocognitive domains. The other studies used a battery of different tests for different domains, and some of these studies also additionally included the MMSE (*n* = 9) or MoCA (*n* = 2) tests. Besides the MMSE, the most commonly used tests were the digit span test (forward test *n* = 13, backward test *n* = 14), which measures short-term memory, the trail making test part A (*n* = 13), which tests processing speed and mental flexibility by connecting numbered dots in sequence, and the digit symbol substitution test (*n* = 11), which measures visuoperceptual functions and motor speed.

Not only the used tests and timing of testing differed considerably between studies, but also the criteria to define a “positive” test result. While some authors use a deterioration compared to baseline testing in terms of the absolute test score (e.g., >2-point deterioration in the MMSE), other authors define a positive test result in terms of the deterioration of ≥1 standard deviation from the baseline measurement. Other authors use *z*-scores, in which the difference in the observed change from baseline between surgical patients and control patients is scaled by the standard deviation of control patients [38]. The “reliable change index” (RCI)—a measure of change from baseline in units of the standard error of the change [39]—has occasionally been used in addition to, or instead of, *z*-scores.

## 4. Discussion

Symptoms of cognitive dysfunction are estimated to occur postoperatively in about 12% of patients without apparent preoperative cognitive dysfunction undergoing noncardiac surgery [4], and the incidence may be as high as 50–70% after cardiac surgery [40]. While these symptoms are often transient, cognitive impairment has been observed in up to 10% of elderly patients at 3 months after surgery [4], and it has been estimated that about one-half of elderly patients with POCD suffer permanent dysfunction [41]. POCD is thus evidently a major threat after an operation in particular to elderly patients, and every effort is needed to prevent, diagnose, and treat POCD in this vulnerable patient population.

Unfortunately, the pathophysiology and risk factors are still poorly understood, and evidence-based treatment options are scarce. It is thus not surprising that POCD is a target of an increasing amount of research papers in anesthesiologic, surgical, and neurologic literature. Using the search term “postoperative cognitive dysfunction” in PubMed yields almost 4,000 results at the time of the writing of this manuscript, with a steady increase over the years: while there were 50 publications in the year 2000, this has increased to 168 in 2010 and to 426 in 2019. The literature, however, has in the past been very heterogenous in how POCD was defined, and this is a major threat to clinical research in this area: when the condition being studied is not well characterized and well defined, study results may not be readily comparable and applicable to clinical practice. Moreover, there are several types of cognitive impairments that can be observed after an operation, including delirium, POCD, and dementia. These terms are sometimes used interchangeably or with a broad overlap in the literature, and a clear distinction is often lacking. In 2010, Rudolph and colleagues have demonstrated a substantial inconsistency in diagnostic criteria, neurocognitive tests, and timing of the assessment of POCD across studies in cardiac surgery [5]. Since then, multiple authors have emphasized the need for uniform terminology and diagnostic criteria in POCD research. However, while it is clear that a uniform approach to defining POCD was previously lacking, it is unclear whether this issue has been successfully improved or even resolved in recent years.

In this focused systematic review, we therefore aimed to study how POCD is defined and measured in the recent literature. The results suggest that even in the most recent POCD literature, a consistent approach towards defining and measuring POCD is still lacking. As previously noted by other authors [4, 42, 43], uniform diagnostic criteria are needed, and this is clearly reinforced by our data.

While our systematic review identified the lack of a consistent approach towards defining POCD, the data, however, provide more than a mere description of heterogeneity across recent studies. We observed some patterns that may serve as a common denominator in the process of reaching standardized diagnostic criteria: with respect to timing, a follow-up measurement at 7 days postoperatively seems to be broadly accepted. With respect to the tests being used, the MMSE, digit span test, trail making test part A, and the digit symbol substitution test were used most commonly, suggesting a broad consensus that these tests are particularly useful for the diagnosis of postoperative neurocognitive disorders. However, it is still unclear which test result would be considered “positive” for POCD. Moreover, it should be noted that these tests have not been developed for POCD testing and have also not been rigorously validated for this purpose. For example, the MMSE has been shown to be less sensitive than the MoCA for a variety of neurocognitive disorders [44], and this could also be true for POCD. This implies that more research not only on the pathophysiology, prevention, and treatment of POCD is required, but also on the diagnostic accuracy [45] of the tests themselves that are commonly used to diagnose POCD.

Our data suggest that strong efforts are necessary to define precise and applicable diagnostic criteria for POCD, involving key-opinion leaders and researchers in the field. This would, in our opinion, be a strong and necessary step towards a precise characterization of the disease, its underlying pathophysiology, risk factors, and treatment options. Notably, however, perhaps the term POCD itself is too broad and vague and actually represents several distinct neuropathologic conditions, rather than one underlying common pathophysiology. If so, this may have important implications for the identification of risk factors as well as prevention and treatment strategies and may explain the heterogeneity in findings across the literature. In this context, Evered et al. have previously suggested that the overarching term POCD should be changed to “delayed neurocognitive recovery” for symptoms expected to have been resolved before 30 days and “postoperative mild neurocognitive disorder” or “postoperative major neurocognitive disorder” for an expected recovery between 30 days and 12 months, depending on the severity of symptoms [6]. Therefore, future efforts should perhaps not so much focus on defining diagnostic criteria for the overarching concept of “POCD” itself, but rather more specifically, for distinct postoperative neurocognitive disorders comprised within this umbrella term. Moreover, it is also well possible that some cognitive domains may be more vulnerable after surgery and anesthesia than others, or that a cognitive decline in some domains may affect patients more severely than a decline in other domains. We believe that identification of those domains that are particularly relevant in the context of POCD is an important topic for future research because broad testing for a decline in all cognitive domains may neither be necessary nor ideal. Focusing on those domains which are most vulnerable and/or most severely affect the patients' wellbeing would reduce the patients' burden to undergo a whole battery of neurocognitive tests, facilitate clinical testing, and allow for a more efficient allocation of clinical and research resources.

The results of this systematic review must be viewed in the context of its limitations. First, this was a focused review, deliberately limited to the literature published in the last two years, and it thus does not claim to be an exhaustive review of all published literature on the topic. However, we specifically attempted to assess how POCD is currently diagnosed, and older literature would merely have been of historic interest. We only searched one database, however, one with comprehensive coverage of reputable medical literature. It was neither our intention to identify gray literature on the topic, nor to identify literature published in predatory or otherwise untrustworthy literature. We are also aware that the mere fact that a majority of studies used a certain definition to diagnose POCD does not necessarily mean that this is the optimal way to diagnose POCD. It does, however, suggest that there is a rather broad consensus among researchers that certain tests at certain time points may play a useful role and can serve as a starting point towards a more uniform definition and assessment of POCD in clinical practice and in research settings.

In conclusion, this systematic review identified the lack of a consistent approach towards defining POCD. Commonalities were identified which may serve as a common denominator for deriving consensus based diagnostic guidelines for POCD. However, more research is necessary to characterize the diagnostic accuracy of the tests used to identify a postoperative cognitive decline and on what would constitute a “positive” test result. Finally, future efforts should perhaps not so much focus on finding a uniform definition of POCD itself, but rather on characterizing and defining distinct postoperative neurocognitive disorders comprised within this overarching term.

## Figures and Tables

**Table 1:** Characteristics of studies reporting diagnostic criteria to define POCD.

Author (year)	Study type/topic	Study population(s)	Type of surgery	POCD criteria	POCD assessment	Timepoint of assessment
Claes et al., 2018 [7]	Prospective cohort study Incidence POCD cochlear implantation	26 (16 male, 10 female) ≥55 years POCD incidence 7 days post-op: 11.5%	Cochlear implantation	A *z*-score of change in MoCA scores ≥1.96.	(i) MoCA	(i) Baseline: (ii) Day of surgery (iii) Post-op: (iv) 7 days
Duan et al., 2018 [8]	Prospective observational study Glial cell line derived neurotropic factor as predictor for POCD	80 (36 male/44 female) 20 healthy controls >50 years POCD incidence 7 days post-op: 47.5%	Elective heart valve replacement	At least 2 *z* scores >1.96. Compared to 20 healthy controls matched by gender, age, and education.	(i) MMSE (ii) Digit span test (iii) Trail making test A (iv) Digit symbol (v) Modalities clock drawing test	(i) Baseline: (ii) 1 day before surgery (iii) Post-op: (iv) 7 days
Gong et al., 2018 [9]	Cohort study Incidence of POCD laparoscopic vs. open surgery	70 (41 male/29 female) 32–85 years POCD incidence 7 days post-op: Open: 18.7% Laparoscopic: 12.5%	Abdominal surgery	Patients with an MMSE score reduction of two.	(i) MMSE	(i) Baseline: (ii) 1 day before (iii) Post-op: (iv) 1 and 7 days
Hayashi et al., 2018 [10]	Secondary analysis 6-minute walk distance and POCD	181 (121 male/60 female) ≥ 60 years POCD incidence 14 days post-op: 28%	Cardiac surgery	Significant decline in cognitive function based on a decrease of 2 or more in the MMSE score.	(i) MMSE	(i) Baseline: (ii) Admission (iii) Post-op: (iv) 14 days
Hou et al., 2018 [11]	RCT Deep versus light anesthesia and POCD	60 (26 male/34 female) ≥60 years POCD incidence 1 day post-op: Low doses: 20% High doses: 3%	Total knee replacement	*A z*-score of >1.96 using cross‐reference.	(i) MoCA	(i) Baseline: (ii) Day of surgery (iii) Post-op: (iv) 1, 3, and 7 days
Konishi et al., 2018 [12]	Secondary analysis of prospective observational study (ACE study) Incidence of POCD sevoflurane vs. propofol	121 sevoflurane (77 male/44 female), 171 propofol (114 male/57 female) ≥60 years POCD incidence 7 days post-op: Sevoflurane: 20.2% Propofol: 15%	Total hip replacement	≥2 tests were at least 1.96 SD lower than the mean score of the matched control group after adjusting for expected change over time using controls or the combined *z-*score was less than −1.96.	(i) Auditory verbal learning test (ii) Trail making test (parts A and B) (iii) Digit symbol substitution test (iv) Controlled oral word association test (v) CERAD semantic fluency test (animals) (vi) Grooved pegboard test (both hands)	(i) Baseline: (ii) The week before surgery (iii) Post-op: (iv) 7 days 3, 12 months
Kumpaitiene et al., 2018 [13]	Prospective observational study POCD and decreased cerebral oxygen saturation	59 (32 male/24 female) ≥60 years POCD incidence 7–10 days post-op: 37%	Coronary artery bypass graft surgery	The sum of all *z*-scores was >2 or at least two *z*-scores for separate tests were >2.	(i) MMSE (ii) Rey auditory verbal learning test (iii) Digit symbol substitution test and Schulte table (iv) Digit span test (v) Trail making test (parts A and B)	(i) Baseline: (ii) Day before surgery (iii) Post-op: (iv) 7–10 days
Şahan et al., 2018 [14]	Pilot study Cerebral oxygen changes	40 (34 male/6 female) ≥60 years POCD incidence 7 days post-op: 45% in target group 41% in control group	Coronary surgery	A drop of 1 standard deviation from baseline on two or more neuropsychological tests.	(i) Logical memory subtest (ii) Clock drawing test Visuospatial skill test (iii) Word list generation test (iv) Digit span subtest	(i) Baseline: (ii) 2-3 days before surgery (iii) Post-op: 7 days (iv) 3 months
Zhang et al., 2018 [15]	Case control study Goal-directed fluid therapy	60 (40 male/20 female) ≥60 POCD incidence 21.67%	Spinal stenosis surgery	If one standard deviation of MoCA score was decreased one or more after surgery.	(i) MoCA	(i) Baseline: (ii) 1 day before surgery (iii) Post-op: (iv) 1, 3, and 7 days
Zhang et al., 2018 [16]	Cohort study Propofol versus sevoflurane and cognitive function	379 (263 male/116 female) ≥ 65 and <90 years POCD incidence 7 days post-op: Sevoflurane: 23.2% propofol: 19.0%	Major cancer surgery (≥2 hours)	Two *z* scores in individual tests or the combined *z* score ≤1.96.	(i) Mental control (ii) Paired associate verbal learning (iii) Grooved pegboard (both hands) (iv) Visual retention (v) Digit symbol (vi) Trail making (part A) (vii) Digit span (forward and backward)	(i) Baseline: (ii) Before surgery (iii) Post-op: (iv) 7 days
Zhang et al., 2018 [17]	Predefined exploratory subanalysis Vitamin D deficiency and POCD	123 (81 male/42 female) ≥65 years POCD incidence 7 days post-op: 24.4%	Major cancer surgery	Two or more *z*-scores in individual tests or the combined *z*-score ≤−1.96.	(i) MMSE Mental control and digit span (forward and backward) (ii) Visual retention and paired associate verbal learning (iii) Digit symbol subtest (iv) Halstead–Reitan trail making test (part A) (v) Grooved pegboard test	(i) Baseline: (ii) Day before surgery (iii) Post-op: (iv) 7 days (v) Telephone: (vi) 30 days post-op
Cheng et al., 2019 [18]	Cohort study Dexmedetomidine versus saline placebo and POCD	535 (392 male/143 female) 100 patients with chronic gastroenteritis (reference normal values) ≥65 years POCD incidence 7 days post-op: Saline: 18% Dexmedetomidine: 12%	Abdominal surgery	RCI ≤−1.96 or a *z*-score ≤−1.96 in at least two tests.	(i) Trail making test (parts A and B) (ii) Digit symbol test (iii) Brief visuospatial memory test-revised (iv) Immediate recall and delayed recall (v) Hopkins verbal learning test-revised (vi) Forward and backward digit span test	(i) Baseline: (ii) Day before surgery (iii) Post-op: (iv) Days 3 and 7 (iv) Telephone: (v) After 1, 3, and 6 months
Daiello et al., 2019 [19]	Secondary analysis of an observational study Association between POCD and postoperative delirium	551 (231 male/320 female) >65 years Incidence POCD 1-month post-op: 47%	Major noncardiac surgery	A composite *z*-score of at least 1.96 across all tests, or *z*-scores for ≥2 tests scores at least 1.96.	(i) Rey visual verbal learning test (ii) Concept shifting test (iii) Stroop color-word test (iv) Letter digit substitution	(i) Baseline: (ii) Within 2 weeks before surgery (iii) Post-op: (iv) 1, 2, and 6 months
Gao et al., 2019 [20]	Prospective study Risk factors of POCD	257 (male 108/149 female) ≥65 years POCD incidence 7 days post-op: 21.4%	Elective total joint arthroplasty	A *z* score >1.96 at least 2 times.	(i) MMSE (ii) Word recognition, memory tests (iii) Digit span test (iv) Verbal fluency test (v) Trail making test (part A) (vi) Symbol digit test	(i) Baseline: (ii) 1 day before surgery (iii) Post-op: (iv) 7 days
Han et al., 2019 [21]	Cohort study Cortisol ratio in saliva as predictor for POCD	94 (65 male/29 female) >60 years POCD incidence 7 days post-op: 17%	Major cardiac surgery	A *z*-score of ≤−1.96 on at least 2 different tests	(i) The short story module of the Randt memory (ii) Grooved pegboard test (dominant and nondominant) (iii) Digit symbol subtest (iv) Trail making test (part A) (v) The verbal fluency test (vi) Digit span (forward and backward) (vii) Finger tapping (viii) Block subtest	(i) Baseline: (ii) 1 day before surgery (iii) Post-op: (iv) 7 days
Holmgaard et al., 2019 [22]	Secondary analysis of a randomized trial Cerebral oximetry and POCD	153 (138 male/15 female) ≥55 years POCD incidence: 29% at discharge, 8% after 3 months	Cardiac surgery (heart valve surgery, coronary artery bypass grafting, or both)	Two out of seven *z*-scores for individual tests or the composite *z*-score >1.96.	(i) MMSE (≤24 excluded) (ii) Visual verbal learning test (iii) Concept shifting test (iv) Stroop color word interference (SCWI) test (v) Letter digit coding (LCD) test	(i) Baseline: (ii) Before surgery (iii) Post-op: (iv) Day before discharge/after 8 days (v) 2–4 months
Hongyu et al., 2019 [23]	Cohort study Effect of penehyclidine hydrochloride on POCD	90 (47 male/43 female) >60 years POCD incidence 7 days post-op: A 53.3% (penehyclidine hydrochloride) B 26.7% (atropine) C 13.3% (saline)	Thoracoscopic surgery for lung cancer	Uneducated <17 points, primary education <20 points, and >6 years education <24 points.	(i) MMSE	(i) Baseline: (ii) 1 day before surgery (iii) Post-op: (iv) 1, 4, and 7 days
Kristek et al., 2019 [24]	Prospective RCT Incidence of POCD effect levobupivacaine versus morphine	70 (4 male/66 female) ≥65 years POCD incidence day 1–discharge: levobupivacaine: 9% Morphine: 31%	Femoral fracture fixation	A decline in the MMSE score below 17 or a reduction in the MMSE score for ≥3 compared with the baseline on at least one measurement.	(i) MMSE	(i) Baseline: (ii) Before surgery (iii) Post-op: (iv) Days 1–5 (v) Day of discharge
Lachmann et al., 2019 [25]	Prospective, observational study Association between cerebral microbleeds and POCD	65 (30 male/35 female) >65 years Incidence POCD 7 days post-op: 20%	Major elective surgery (>60 minutes)	RCI by Rasmussen et al., 2001.	(i) Paired associates learning (ii) Verbal recognition memory (iii) Spatial span time (iv) Simple reaction time (v) Grooved pegboard (vi) Trail making tests	(i) Baseline: (ii) Performed but not mentioned when. (iii) Post-op: (iv) 7 days (v) 3 months
Langer et al., 2019 [26]	RCT pilot Hypotension and POCD	101 patients (53 male/ 48 female) 33 age-matched controls ≥75 years POCD incidence 3 months post-op: 9%	Elective noncardiac surgery	RCI was used A *z*-score below −1.96 on at least 2 neuropsychological tests and/or the combined *z*-score below −1.96.	(i) MMSE (ii) Stroop test (iii) Symbol digit modalities test (iv) Trail making test (parts A and B) (v) Immediate and delayed recall (vi) Free and cued selective reminding test (FCSRT) (vii) Verbal phonemic fluency test (viii) Denomination test	(i) Baseline: (ii) Before surgery (iii) Post-op: (iv) After 3 months
Li et al., 2019 [27]	Prospective, observational study Association between serum levels S100A12 and POCD	186 patients (male 78/female 108) 186 controls (for serum) ≥65 years POCD incidence 7 days post-op: 35%	Femoral neck fracture or intertrochanteric fracture	Two or more *z*-scores in individual tests or the combined *z*-score ≤−1.96.	(i) Visual verbal learning test (ii) Concept shifting test (iii) Stroop color word interference test (iv) Paper and pencil memory scanning test (v) Letter digit coding (vi) Four boxes test	(i) Baseline: (ii) 1 day before surgery (iii) Post-op: (iv) 7 days
Li et al., 2019 [28]	Prospective RCT preliminary trial Sedation by dexmedetomidine, midazolam, or propofol and POCD	205 (72 male/133 female) ≥65 years POCD incidence 7 days post-op: 36.6%	Hip or knee arthroplasty	RCI score less than −1.96 on ≥2 tests and/or combined *z*-score less than −1.96.	(i) Montreal cognitive assessment (MoCA) (ii) Stroop color-word test (SCWT) (iii) Associative learning and memory test (iv) Digit symbol test (v) Digit span test	(i) Baseline: (ii) Before surgery (iii) Post-op: (iv) 7 days (v) Telephone: (vi) 1 year using 5-minute MoCA protocol
Quan et al., 2019 [29]	RCT Light vs. deep anesthesia and POCD	120 (66 male/54 female) ≥60 years POCD incidence 7 days post-op: Deep: 19.2% Light: 39.6%	Abdominal surgery	Deterioration in postoperative performance by 1 or more standard deviations on 2 or more tests.	(i) Mental control (ii) Digit span (backward and forward) (iii) Paired associate verbal learning (iv) Digit symbol subtest (v) Visual retention-Halstead–Reitan trail making test (part A)-grooved pegboard test	(i) Baseline: (ii) Before surgery (iii) Post-op: (iv) 7 days (v) 3 months
Sánchez et al., 2019 [30]	Longitudinal study Optimization of delirium assessment	1500 ≥70 years POCD incidence:	Elective surgery Thoracic, cardiac, vascular, proximal big joints and spine, gastrointestinal, genitourinary, and general elective surgery	A test value ≤0.5 standard deviations, normalized for age, gender, and education, in one of these tests.	(i) Montreal cognitive assessment (MoCA) (ii) Digit span backwards (iii) Trail making test A and B	(i) Baseline: (ii) Preadmission (iii) Before surgery (iv) Post-op: (v) 2, 6, and 12 months
Wang et al., 2019 [31]	Prospective cohort study Glucocorticoid receptor, FKBP51, and POCD in elderly	111 (49 male/62 female) ≥65 years POCD incidence 7 days post-op: 28.3%	Hip fracture surgery	A decline of 1 or more standard deviations (SDs) in 2 or more tests.	(i) MMSE (ii) Verbal learning (iii) Mental control (iv) Digit span (forward and backward) (v) Visual retention (vi) Grooved pegboard test (both hands) (vii) Digit symbol test (viii) Trail making test (part A)	(i) Baseline: (ii) 1 day before surgery (iii) Post-op: (iv) 7 days
Wang et al., 2019 [32]	Cohort study circRNA_089763 as biomarker for POCD	35 (30 male/5 female) 40–90 years POCD incidence 7 days post-op: 34%	Coronary artery bypass grafting	Reduction in postoperative test score compared with the preoperative test score ≥20% in ≥2 tests.	(i) Stroop color and word test (ii) Trail making test (iii) Digit symbol substitution test (iv) Verbal learning test (v) The symbol digits modalities test	(i) Baseline: (ii) 1 day before surgery (iii) Post-op: (iv) 7 days
Wang et al., 2019 [33]	Prospective observational cohort study Smoking history and risk for POCD	382 (189 male/193 female) ≥60 years POCD incidence 5 days post-op: 50.4%	Noncardiac surgery	A *z*-score of ≥1.96.	(i) MMSE (ii) Concept shifting test (iii) Stroop color-word interference test	(i) Baseline: (ii) 1 day before surgery (iii) Post-op: (iv) 5 and 7 days
Wang et al., 2019 [34]	RCT Ropivacaine intercostal nerve block and POCD	100 (50 male/50 female) Age: 43–46 years POCD incidence: With block: 21% Without block: 39%	Thoracotomy for esophageal cancer	Score less than or equal to 25 points, or if more than 2 points reduction on the MMSE score after surgery compared with the score before surgery.	(i) MMSE	(i) Baseline: (ii) 1 hour before surgery (iii) Post-op: (iv) 2, 12, and 24 hours
Zhang et al., 2019 [35]	Cohort study Effects of fentanyl versus sufentanil on POCD	96 (47 male/49 female) ≥65 years POCD incidence 7 days post-op: Fentanyl: 11.9% Sufentanil: 6.2%	Open surgery	A drop of 1 standard deviation from baseline on two or more items.	(i) MoCA	(i) Baseline: (ii) 1 day before surgery (iii) Post-op: (iv) 1, 7 days
Zhang et al., 2019 [36]	Cohort study Risk factors for POCD	77 (42 male/35 female) ≥65 years POCD 7 days post-op: 24.7%	Colorectal surgery	*z*-score was greater than 1.96 or the combined *z*- score was ≥1.96.	(i) MMSE (ii) Visual verbal learning test (iii) Digit span test (iv) Digit symbol substitution test	(i) Baseline: (ii) 1 day before surgery (iii) Post-op: (iv) 7 days

**Table 2:** Neurocognitive tests used in the included studies to define POCD.

Neurocognitive test	Number of studies, in which the test was used
Associative learning and memory test	1
Auditory verbal learning test Block subtest	2 1
Clock drawing test	2
Concept shifting test	4
Controlled oral word association test	1
Denomination test	1
Digit symbol substitution test	11
Digit span test Forward Backward Finger tapping test	13 14 1
Four boxes test	1
Free and cued selective reminding test	1
Grooved pegboard test	7
Hopkins verbal learning test	1
Immediate recall and delayed recall	2
Letter digit coding test Letter digit substitution test	2 1
Logical memory test	1
Mental control	4
MMSE	14
MoCA	6
Paired association verbal learning test	5
Paper pencil memory scanning test	1
Stroop color interference test Short story module of the Randt memory Simple reaction time Spatial span time Symbol digits modalities test	7 1 1 1 1
Trail making test Part A Part B	13 6
Verbal fluency test	3
Verbal learning test	4
Visuospatial memory test (brief)	1
Visuospatial skill test	1
Visual retention	4
Visual verbal learning test (here: with capital letters)	4
Word list generation test	1
Word recognition memory test	1

## Data Availability

All data are extracted from the published literature, which are available from the respective publisher upon request.
